# Conjunctival Rhinosporidosis Mimicking Papilloma in an Ethiopian Child: A Rare Case Report

**DOI:** 10.1002/ccr3.70439

**Published:** 2025-05-05

**Authors:** Seblewengel Maru Wubalem, Social Beyecaha Diro

**Affiliations:** ^1^ Department of Pathology Wachemo University Hossana Ethiopia; ^2^ Department of Ophthalmology Hawassa University Comprhesnsive Specialized Hospital Tertiary Eye Center Hawassa Ethiopia

**Keywords:** conjunctiva, eye, ocular rhinosporidiosis, papilloma

## Abstract

Rhinosporidiosis is a chronic granulomatous disease caused by Rhinosporidium seeberi, mainly affecting the nose and nasopharynx. We report a sporadic occurrence of conjunctival rhinosporidiosis that mimicked papilloma in a 10‐year‐old Ethiopian child. Rhinosporidiosis should be considered in the differential diagnosis of conjunctival lesions. Histopathology is necessary for a definitive diagnosis.

## 1 Introduction

Rhinosporidiosis is a chronic granulomatous disease of the mucous membranes caused by Rhinosporidium seeberi, a microorganism categorized under the class Mesomycetozoea [1, 2]. It has the highest incidence in India and Sri Lanka [3] and is rarely seen in Africa [1, 4]. Men are predominantly affected compared to women, and the disease occurs in both adults and children [5]. Nasal and nasopharyngeal involvement is the most common presentation, observed in 70% of cases, followed by ocular lesions seen in 15%. Other rare sites of involvement include the lips, palate, uvula, maxillary antrum, epiglottis, larynx, pharynx, trachea/bronchi, and ear [3].

The probable mode of transmission of R. seeberi is through the injured epithelium of the nasal mucosa, conjunctiva, or skin that comes into contact with contaminated water or soil [2, 3, 6, 7]. Another suggested mode of infection in dry regions is the inhalation of field dust contaminated by spores [3, 8]. In this report, we present a rare case of conjunctival rhinosporidiosis in a 10‐year‐old Ethiopian child.

## 2 Case History/Examination

A 10‐year‐old male patient presented with a mass around the eyelid, experiencing a foreign body sensation, discharge, redness, and irritation in the right eye for a duration of 2 weeks. He reported no history of eye pain, reduction in vision, or double vision, and denied any history of eye trauma. Additionally, he denied any photophobia or eye fatigue. The patient has no significant medical history, has never applied medication to his eye, and has no known allergies. He has not undergone any previous eye surgeries. There is no family history of eye conditions, and he has not experienced similar symptoms in the past.

Ophthalmologic evaluation revealed a visual acuity of 20/20 in both eyes. Intraocular pressure was 13 mmHg in the right eye and 14 mmHg in the left eye. Upon examination, the eyelid, bulbar conjunctiva, sclera, and cornea all appeared normal. A visible mass was observed hanging over the margin of the right lower lid, covered by discharge. After cleansing the discharge and everting the right lower lid, a pedunculated, fleshy, and vascularized mass originating from the lower conjunctival fornix was identified. Ocular motility and posterior segment findings were normal.

## 3 Methods

The clinical impression was a pedunculated papilloma of the right lower foniceal conjunctiva, and an excisional biopsy without cauterization was performed. There were no complications during or after the procedure. The patient was sent home the same day as the procedure. The specimen was sent for pathologic examination.

**FIGURE 1:**
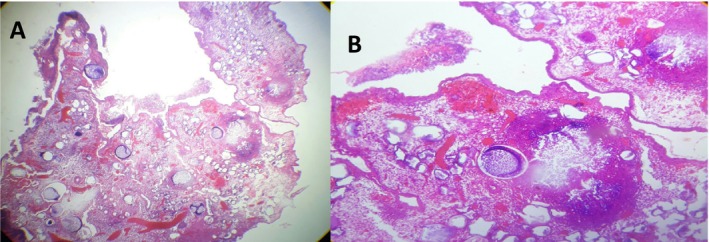
Histopathology revealed multiple intact and ruptured thick‐walled sporangia containing endospores, with surrounding loose fibrous stroma, abundant variably sized thin‐walled blood vessels, (Hematoxilin and Eosin, A 40×, B 200×).

**FIGURE 2:**
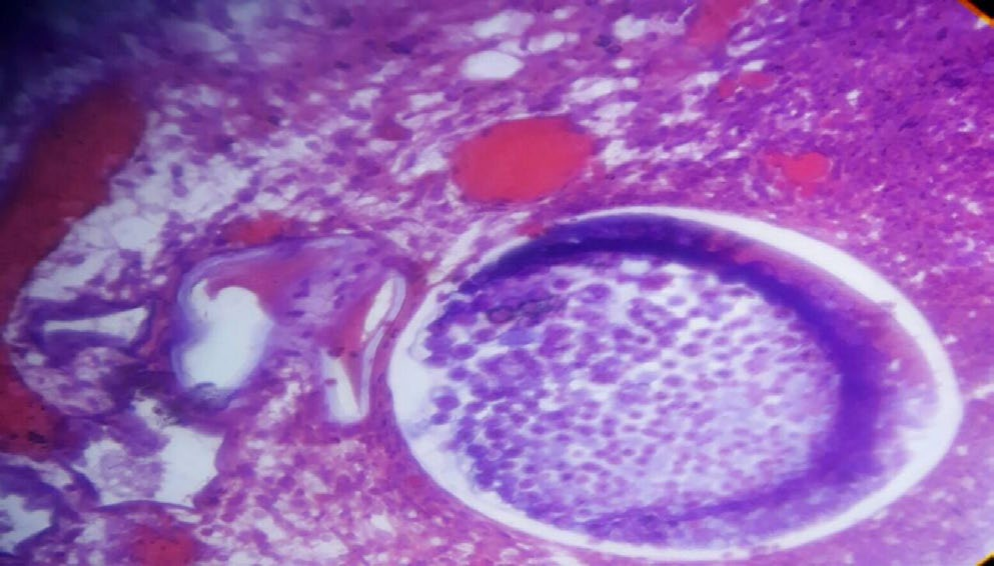
Histopathologic section showing thick‐walled sporangia containing endospores formation (Hematoxilin and Eosin, 400×).

Grossly, a 1 × 0.5 cm gray‐white ovoid tissue was received. The histopathologic examination revealed ulcerated stratified squamous epithelium lining. Underneath, there were multiple intact and ruptured thick‐walled sporangia containing endospores, with surrounding loose fibrous stroma, abundant variably sized thin‐walled blood vessels, and lymphoplasmacytic infiltrates (Figures 1 and 2). Subsequently, a diagnosis of conjunctival rhinosporidiosis was made.

## 4 Conclusions and Results (Outcome and Follow‐Up)

The patient has been regularly followed for 10 months, and there have not been any signs of recurrence.

## 5 Discussion

Ocular rhinosporidiosis accounts for 15% of all rhinosporidial infections [3]. The conjunctiva is the most commonly affected ocular structure, in 50%–90% of cases, followed by the lacrimal sac and eyelid [9, 10, 11]. One case of orbital involvement was also reported in a study conducted by Alam et al. [10].

Ocular rhinosporidiosis typically presents as a friable, highly vascularized, pedunculated, polypoid mass arising from the palpebral conjunctiva. When it arises from the bulbar conjunctiva, it appears sessile or flat, as there is no space to grow due to the pressure exerted by the eyelids over the conjunctiva [7]. It usually has yellow spots on the surface that correspond to mature sporangia [4, 7, 11]. These features are important indicators for differentiating rhinosporidiosis from other differential diagnoses. Other presentations include diverticulum of the lacrimal sac, recurrent chalazion, cysts, or chronic follicular reaction of the conjunctiva, especially in contact lens users, as well as keratitis, scleral melting, ciliary staphyloma, bleeding, or eyelid tumors [2, 11]. Painful proptosis is the presenting feature in cases of orbital involvement [10].

Diagnosis of rhinosporidiosis by Fine Needle Aspiration Cytology can be helpful, especially in resource‐limited setups. However, the pathognomonic test for rhinosporidiosis is the histopathologic examination of the resected specimen. Grossly, Rhinosporidium seeberi presents as rounded structures that can be seen as yellowish, pinhead‐sized spots on the surface of the polyp. Microscopically, the presence of numerous sporangia at varying stages of maturation filled with endospores confirms the diagnosis. These structures vary in size, corresponding to various stages of development of the organism. The mature stage of sporangia consists of a large, thick, and spherical wall that encloses smaller daughter cells named endospores. Special stains, such as Grocott‐Gomori's methenamine silver, periodic acid–Schiff, and mucicarmine, can be used to enhance the microscopic features of these structures. The lesions usually have associated granulomatous inflammation, fibrosis, and granulation tissue [3, 12]. In our case, the definitive diagnosis was made by observing multiple intact and ruptured thick‐walled sporangia containing endospores in hematoxylin and eosin‐stained histological sections.

Surgical therapy is the mainstay of treatment for ocular rhinosporidiosis, with a good prognosis [9, 11]. Cauterization of the base following surgical excision significantly decreases the rate of recurrence [10, 11]. The case series of 12 patients by Adhik et al., who were managed with surgical excision and cauterization of the base, shows no recurrence in any of the patients after 1 year of follow‐up [11]. For cases involving the lacrimal sac, dacryocystectomy is recommended. Dacryocystectomy should be performed with caution to avoid spilling the spores, and complete excision of the intact sac is necessary to minimize recurrence rates [9]. Medical treatment of the disease is not as effective as surgical management, since cultures of R. seeberi have been unsuccessful in all artificial media, making sensitivity determination difficult. Dapsone has been implicated to have some benefit by arresting the maturation of sporangia and accelerating their degeneration [8, 9, 11, 13].

Clinically, conjunctival rhinosporidiosis can mimic other pathologies, such as papilloma, pyogenic granuloma, and eye tumors [2, 10]. A high level of clinical suspicion is essential for differentiation, particularly in sporadic regions such as Ethiopia. Analyzing the lesion for yellowish spots on its surface can provide hints toward conjunctival rhinosporidiosis. However, definitive differentiation is achieved through histopathological examination. In the present case, it was clinically considered to be a papilloma. There are three case reports of conjunctival rhinosporidiosis from Ethiopia, and in all of them, as well as in the current case, ocular rhinosporidiosis was not entertained clinically, highlighting its rarity and the possibility of mimicking other disease entities [14, 15, 16].

## 6 Conclusion

Ocular rhinosporidosis is a rare infection in regions like Ethiopia where it is not endemic. This report aims to inform ophthalmologists and pathologists about the possibility of sporadic conjunctival rhinosporidosis. Consequently, it should be included in the differential diagnosis of polypoid conjunctival lesions. A histopathological examination is required for a definitive diagnosis.

## Author Contributions

**Seblewengel Maru Wubalem:** conceptualization, data curation, resources, supervision, visualization, writing – original draft, writing – review and editing. **Social Beyecaha Diro:** data curation, visualization, writing – original draft, writing – review and editing.

## Consent

Written informed consent for publication of this case report and accompanying images was obtained from the patient's parent.

## Conflicts of Interest

The authors declare no conflicts of interest.

## Data Availability

Data sharing not applicable to this article as no datasets were generated or analyzed during the current study.
